# *Pseudomonas aeruginosa* Utilizes the Type III Secreted Toxin ExoS to Avoid Acidified Compartments within Epithelial Cells

**DOI:** 10.1371/journal.pone.0073111

**Published:** 2013-09-18

**Authors:** Susan R. Heimer, David J. Evans, Michael E. Stern, Joseph T. Barbieri, Timothy Yahr, Suzanne M. J. Fleiszig

**Affiliations:** 1 School of Optometry, University of California, Berkeley, California, United States of America; 2 College of Pharmacy, Touro University California, Vallejo, California, United States of America; 3 Allergan, Inc., Irvine, California, United States of America; 4 Microbiology and Molecular Genetics, Medical College of Wisconsin, Milwaukee, Wisconsin, United States of America; 5 Department of Microbiology, University of Iowa, Iowa City, Iowa, United States of America; 6 Graduate Groups in Vision Sciences, Microbiology and Infectious Diseases & Immunity, University of California, Berkeley, California, United States of America; The Scripps Research Institute and Sorrento Therapeutics, Inc., United States of America

## Abstract

Invasive *Pseudomonas aeruginosa* (PA) can enter epithelial cells wherein they mediate formation of plasma membrane bleb-niches for intracellular compartmentalization. This phenotype, and capacity for intracellular replication, requires the ADP-ribosyltransferase (ADPr) activity of ExoS, a PA type III secretion system (T3SS) effector protein. Thus, PA T3SS mutants lack these capacities and instead traffic to perinuclear vacuoles. Here, we tested the hypothesis that the T3SS, via the ADPr activity of ExoS, allows PA to evade acidic vacuoles that otherwise suppress its intracellular viability. The acidification state of bacteria-occupied vacuoles within infected corneal epithelial cells was studied using LysoTracker to visualize acidic, lysosomal vacuoles. Steady state analysis showed that within cells wild-type PAO1 localized to both membrane bleb-niches and vacuoles, while both *exsA* (transcriptional activator) and *popB* (effector translocation) T3SS mutants were only found in vacuoles. The acidification state of occupied vacuoles suggested a relationship with ExoS expression, i.e. vacuoles occupied by the *exsA* mutant (unable to express ExoS) were more often acidified than either *popB* mutant or wild-type PAO1 occupied vacuoles (p < 0.001). An *exoS-gfp* reporter construct pJNE05 confirmed that high *exoS* transcriptional output coincided with low occupation of acidified vacuoles, and *vice versa*, for both *popB* mutants and wild-type bacteria. Complementation of a triple effector null mutant of PAO1 with *exoS* (pUCP*exoS*) reduced the number of acidified bacteria-occupied vacuoles per cell; pUCP*exoS*E381D which lacks ADPr activity did not. The H^+^-ATPase inhibitor bafilomycin rescued intracellular replication to wild-type levels for *exsA* mutants, showing its viability is suppressed by vacuolar acidification. Taken together, the data show that the mechanism by which ExoS ADPr activity allows intracellular replication by PA involves suppression of vacuolar acidification. They also show that variability in ExoS expression by wild-type PA inside cells can differentially influence the fate of individual intracellular bacteria, even within the same cell.

## Introduction

*Pseudomonas aeruginosa* is a highly adaptable bacterial pathogen that plays a major role in nosocomial infections including pneumonia, septicemia, and urinary tract infections, as well as community-acquired opportunistic infections of the skin, soft tissue, and ocular surface [1-7]. *P. aeruginosa* adaptability is reflected by the diversity of genetic traits and large genome sizes seen among clinical isolates, suggesting it has a proclivity for acquiring new DNA through horizontal transfer and retaining traits that enable survival in different host tissues [8,9]. Part of *P. aeruginosa*’s success as a pathogen is derived from its ability to adapt to the *in vivo* environment, and express virulence traits that help the bacteria evade host defenses. In the latter regard, the type III secretion system (T3SS) plays a major role through the expression of one or more of four known effector proteins ExoS, ExoU, ExoT and ExoY which promote *P. aeruginosa* virulence by modulating bacterial interactions with epithelial cells, immune cells, and host tissues [10-16].

Phagocytes and some "non-professional" phagocytes, including epithelial cells, facilitate the destruction of internalized microbes by trafficking them through a series of intracellular vacuolar compartments starting in phagosomes (similar to early endosomes) and terminating in acidified bactericidal phagolysosomes [17]. Some microbes meet a similar fate via autophagy in which autophagosomes fuse with lysosomes to form acidified bactericidal autolysosomes [18]. Successful intracellular pathogens, however, either show intrinsic resistance to acidified phagolysosomes, e.g. *Coxiella* spp. or *Mycobacterium* spp. [19,20] and/or escape default trafficking to establish alternative intracellular survival niches. For example, *Listeria monocytogenes* uses listeriolysin O to destabilize vacuolar membranes and escape to the cytosol [21], and *Streptococcus pyogenes* uses streptolysin O to reduce lysosomal colocalization bacterial-occupied vacuoles [22]. *Burkholderia cenocepacia* containing vacuoles acquire late endosomal markers, but delay recruitment of the NADPH oxidase needed for vacuole acidification using type 6 secretion system-dependent interference with RhoGTPases [23,24]. Other Gram-negative bacteria utilize a T3SS to survive intracellularly. These include *Salmonella enterica* altering the maturation of early endosomes by manipulating Rab proteins involved in vacuolar fusion, allowing formation of a *Salmonella* -containing vacuole [25-27], and *Shigella* spp. using a T3SS effector IcsB to escape autophagy in the cytosol [28].

We previously reported that the ADPr activity of the *P. aeruginosa* T3SS effector ExoS promotes *P. aeruginosa* intracellular survival and is associated with the formation of membrane bleb-niches within human epithelial cells [16,29]. Mutants in the T3SS that cannot express ExoS, e.g. *exsA* (T3SS transcriptional activator) mutants and *pscC* (T3SS needle) mutants, or *exoS* mutants lacking ADPr activity, do not induce bleb formation, are defective in intracellular survival, and traffic to perinuclear vacuoles [16,29]. Using *exsA* mutants, we have shown that these perinuclear vacuoles are LAMP3+ [29], a feature of late endosomes. In contrast, *popB* mutants (which lack the T3SS translocon, but can secrete effectors) traffic to LAMP3- vacuoles and retain the capacity to replicate intracellularly. Like wild-type *P. aeruginosa*, replication of *popB* mutants is dependent on the ADPr activity of ExoS [30].

The aim of this study was to further our understanding of how ExoS ADPr activity enables *P. aeruginosa* to replicate intracellularly, and how epithelial cells suppress *P. aeruginosa* viability when ExoS activity is absent. Thus, we tested the hypothesis that ExoS-mediated intracellular survival involves evasion of acidified intracellular compartments, and that without ExoS, internalized bacteria are trafficked to acidified vacuolar compartments wherein they lose viability.

## Materials and Methods

### Bacterial Strains

*P. aeruginosa* strain PAO1, T3SS mutants, and plasmid-complemented strains used in this study are described in Table 1. For fluorescent imaging, bacteria were transformed by electroporation with plasmids encoding either green fluorescent protein (pSMC2) [31] or dTomato (p67T1) [32] and selectively cultured at 37°C overnight on tryptic soy agar (TSA) (BD Bioscience, CA) containing carbenicillin (200 µg/mL) (Sigma, MO). If antibiotic selection was not needed, bacteria were grown on TSA plates at 37°C overnight. Bacterial inocula were prepared by resuspending in warm keratinocyte growth medium (KGM) (no antibiotics) to an optical density of 0.1 at 650 nm (Spectronic 21D; Milton Roy, PA), and diluted 1:10 to yield ~1 x 10^7^ CFU/mL. Inoculum sizes were confirmed by viable count. To study *exoS* transcription, PAO1 and the *popB* mutant were transformed by electroporation with a reporter plasmid, bearing *gfp* under control of the *exoS* promoter (pJNE05) [33], and cultured at 37°C overnight on TSA containing gentamicin (200 µg/mL) (Lonza, MD). Expression of the GFP-reporter was confirmed under T3SS-inducing conditions [Tryptic soy broth supplemented with 1% glycerol, 100 mM monosodium glutamate, and 2 mM EGTA (Sigma, MO)].

**Table 1:** Bacterial strains, mutants and recombinant plasmids used.

Strain, mutant, and/or plasmid	T3SS Description	Replicates in Epithelial cells	Reference
PAO1	Wild-type *P. aeruginosa*. Expresses ExoS, ExoT, ExoY	+	[13,29]
PAO1*exsA*::Ω	Lacks T3S transcriptional activator	-	[29,48]
(*exsA* mutant)	No T3S expression		
PAO1Δ*popB*	Lacks T3S translocon	+	[13,29]
(*popB* mutant)	Encodes ExoS, ExoT, ExoY		
PAO1Δ*exoSTY*	No known T3S effectors	-	[13,29]
PAO1Δ*exoSTY* *+* pUCP*exoS*	Complementation with plasmid-expressed ExoS	+	[16,49]
PAO1Δ*exoSTY* *+* pUCP*exoS* (E381D)	Complementation with plasmid-expressed ExoS without ADPr activity	-	[16,49]
PAO1Δ*exoSTY* *+* pUCP18	Plasmid control	-	[16]
pSMC2	Plasmid encoding constitutively-expressed green fluorescent protein (GFP)	NA	[31]
pJNE05	Plasmid encoding *gfp* fused the ExsA-dependent promoter of *exoS*	NA	[33]
p67T1	Plasmid encoding constitutively expressed dTomato	NA	[32]

### Cell Culture

Telomerase-immortalized human corneal epithelial cells (hTCEpi) [34] were cultured in KGM containing the antibiotics gentamicin (30 µg/mL) and amphotericin B (15 ng/mL) (Lonza, MD) at 37 °C under 5% CO_2_ on sterile 25 mm glass coverslips until ~ 80% confluence. Prior to infection (24 h), cultures were washed with 3 equal volumes (2 mL) of warm phosphate buffered saline (PBS) and switched to KGM without antibiotics.

### Confocal Microscopy

Epithelial cells were inoculated with ~10^7^ CFU/mL of bacteria and incubated for 3 h at 37 °C (5% CO_2_). Viable extracellular bacteria were then eliminated by washing with 3 equivalent volumes (2 mL) of warm PBS and culturing in warm KGM (2 mL) containing amikacin (200 µg/mL) (Sigma, MO) for 1 h at 37 °C (5% CO_2_). Infected cultures were then stained with the acidophilic dye - LysoTracker DND-22 (Life Technologies, NY) as a 1 µM solution in warm, phenol red - free KGM (Promocell, Germany) containing amikacin (200 µg/mL) for 30 min as described above. Cultures were then immediately transferred to an attofluor chamber (Life Technologies, NY) and viewed with a Fluoview FV1000 laser scanning confocal microscope (Olympus, PA) equipped with 60 x magnification water-immersion objective, 100W halogen illumination (for Nomarski differential interference contrast - DIC), 405 nm and 559 nm diode lasers (used for the excitation of LysoTracker DND-22 and dTomato, respectively) and a multi-line argon laser (used to excite GFP at 488 nm). Fluorescent and transmitted light was collected simultaneously using spectral-based PMT detection and integrated DIC in 0.5 µm increments along the z-axis. Resulting images were processed and quantified with FV1000 ASW software (Olympus, PA) using ≥10 fields per condition. Each field contained an average of 10 infected epithelial cells. Thus, for each condition, ~100 infected cells and >300 bacteria-occupied vacuoles were counted or measured respectively. The diameter of each bacteria-occupied vacuole was also noted. Mean values of bacteria-occupied vacuoles per cell and relative percentage of total occupied vacuoles are reported along with standard error of the mean (SEM). In some instances, the mean value of all intracellular bacteria per cell, including bacteria within bleb niches, was also tabulated. Statistical significance was assessed with ANOVA followed by a Welch’s corrected t-Test based on the unequal variance of each normally distributed dataset.

### Intracellular Survival Assays

Bacterial survival and intracellular replication was assessed using culture conditions slightly modified from those described above. Paired sets of epithelial cell cultures were grown in 12 well tissue culture plates to confluence in KGM containing antibiotics (gentamicin and amphotericin B as above) and switched to antibiotic-free media 24 h prior to infection. To block vacuolar acidification, a subset of cultures were treated with a vATPase inhibitor, bafilomycin A1 (Sigma, MO), suspended in KGM (final concentration of 200 nM). These treatments were initiated 1 h prior to infection and maintained throughout the assay. Epithelial cells were inoculated with ~10^6^ CFU/mL of bacteria (in 1 mL) and incubated for 3 h at 37 °C (5% CO_2_). Viable extracellular bacteria were then removed by washing with 3 volumes (2 mL) of warm PBS, then incubating with warm KGM containing amikacin (200 µg/mL), as previously described, for 1 h (4 h time point) or 5 h (8 h time point) amongst paired cultures, to allow intracellular replication. Viable intracellular bacteria were recovered from PBS-washed cultures using a 0.25% Triton X-100 solution (0.5 mL/well) and enumerated by viable counting on TSA plates. Each sample was assessed in triplicate, and data were expressed as a mean +/- SEM per sample. Intracellular replication was reported as the increase in recovered CFU at 8 h post-infection as a percentage of a baseline measurement made after 4 h.

### Statistical Analysis

Significance of differences between groups was assessed using ANOVA and Welch’s corrected t-Test (based on unequal variance among normally distributed datasets) or the Chi-square test. P values < 0.05 were considered significant. Experiments were repeated at least three times unless stated otherwise.

## Results

### *P. aeruginosa* Mutants Lacking Expression of Type III Secretion Traffic to Acidified Vacuoles

**Figure 1:**
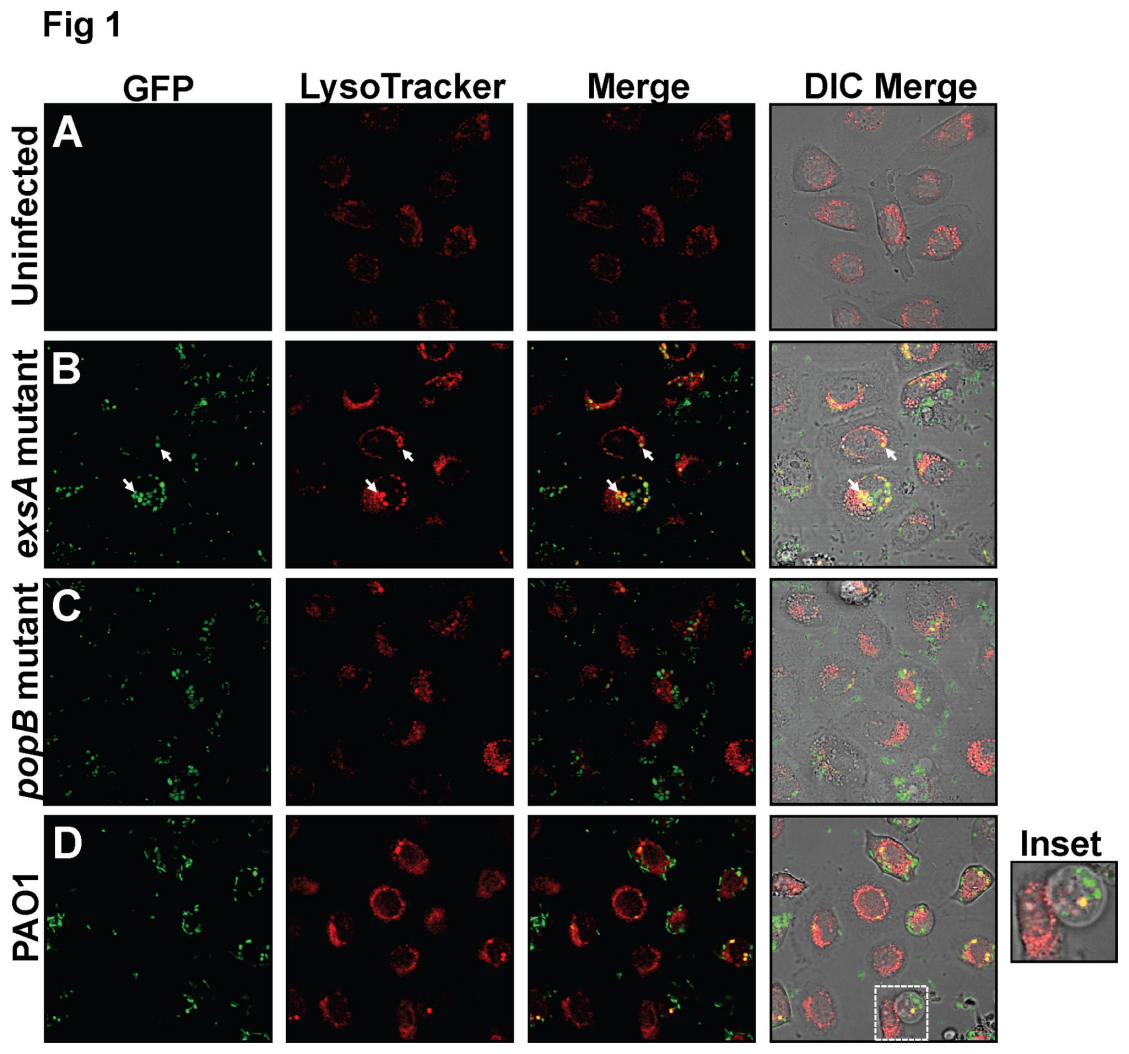
Colocalization of the *P. aeruginosa exsA* mutant with acidified vacuoles in epithelial cells compared to that of wild-type bacteria or a *popB* (translocon) mutant. Confocal microscopy images of human corneal epithelial cells at 5 h post-infection with GFP-expressing *P. aeruginosa* (green). Prior to imaging, infected cultures were infused with LysoTracker (LT) DND-22 (pseudo-colored red). Panels depict (A) Uninfected control, (B) PAO1 *exsA* mutant (C) PAO1 *popB* (translocon) mutant and (D) wild-type PAO1. Uninfected cells appeared healthy. The intracellular *exsA* mutant appeared more frequently in LT (+) (acidified) vacuoles which co-localized yellow (arrows) than either the intracellular *popB* mutant or wild-type PAO1. PAO1-infected cells which displayed bleb-niche formation (1D inset) showed reduced fluorescence (< 10% fluorescence intensity of PAO1-infected non-blebbing cells, p < 0.001 Welch’s corrected t-Test). Occasional bleb-niches contained LT (+) vacuoles containing bacteria (1D inset, yellow). Representative images are shown. Magnification ~ 600 x.

We have previously shown that T3SS (*exsA*) mutants of *P. aeruginosa* strain PAO1 traffic to perinuclear vacuoles that label with the late endosomal marker LAMP3 after they are internalization by epithelial cells, and that this correlates with an inability to thrive [29]. Here, we examined whether vacuoles occupied by *exsA* mutants of PAO1 were acidified. Confocal imaging of human corneal epithelial cells infected with GFP-expressing *P. aeruginosa* and labeled with LysoTracker (LT) DND-22 showed different intracellular localization for wild-type bacteria and T3SS mutants (*exsA* or *popB*) at 5 h post-infection (Figure 1). As expected, intracellular *exsA* mutants were confined to vacuoles, and the majority of these vacuoles were found to be LT-labeled (i.e. acidified) (Figure 1B, co-localization appears yellow). In contrast, translocon (*popB*) mutants and wild-type bacteria, which can both replicate intracellularly (due to their capacity to secrete ExoS), showed little or no co-localization with acidified vacuoles (Figure 1C and 1D, respectively). As expected, wild-type bacteria caused membrane bleb-niche formation in (~50%) of PAO1 infected cells (Figure 1D inset). Interestingly, LT (-) individual bleb niches occasionally contained LT (+) vacuoles containing bacteria (see Figure 1D inset). However, blebbing cells otherwise stained poorly with LT, displaying > 20-fold reduced total fluorescence intensity [174.5 +/- 78] compared to non-blebbing PAO1-infected cells [3737.0 +/- 708.8] [p < 0.001 Welch’s corrected t-Test]. Non-blebbing PAO1-infected cells showed similar intensity to cells infected with the *exsA* mutant [3537.7 +/- 205.9].

**Figure 2:**
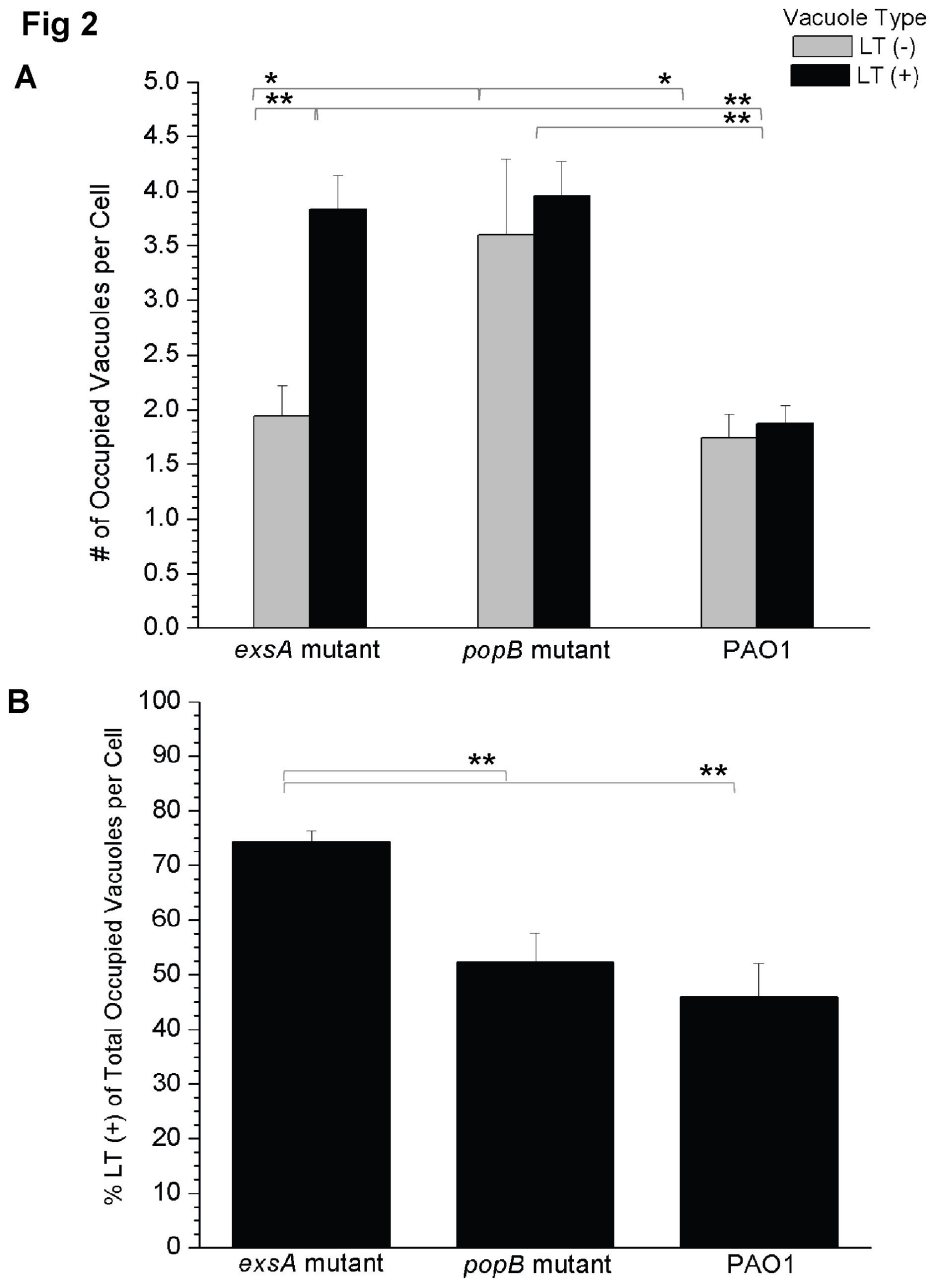
Quantification of acidified versus non-acidified vacuole occupation by wild-type *P. aeruginosa* and its type III secretion mutants. (A) Confocal microscopy images were used to classify bacteria-occupied vacuoles in human corneal epithelial cells as either LT (+) (acidified) or LT (-) (non-acidic) at 5 h post-infection with *P. aeruginosa* PAO1 or its type III secretion mutants (*exsA* or *popB*). The data are shown as the mean (+/- SEM) number of bacteria-occupied vacuoles per cell. Grey columns denote LT (-) vacuoles, black columns LT (+) vacuoles. The *exsA* and *popB* mutants were both associated with increased numbers of acidified LT (+) bacteria-occupied vacuoles per cell compared to wild-type PAO1 (p < 0.001, Welch’s corrected t-Test). The *exsA* mutant showed more acidified than non-acidified bacteria-occupied vacuoles per cell (p < 0.001, Welch’s corrected t-Test). (B) To normalize differences in internalization and replication, the percentage of LT (+) bacteria-occupied vacuoles was calculated as a function of the total number of bacteria-occupied vacuoles per cell. Mean percentage (+/- SEM) is shown. The *exsA* mutant was associated with more acidified bacteria-occupied vacuoles per cell than either the *popB* mutant or wild-type bacteria (p < 0.001, Welch’s corrected t-Test). A representative experiment of 3 independent experiments is shown in both panels (A) and (B). Calculations excluded cells showing bleb-niche formation. Significant differences between all groups were identified using ANOVA analysis (p < 0.0001), and characterized on a pairwise basis using Welch’s correct t-Test [*p < 0.05, **p < 0.001].

The number of vacuoles per cell occupied by *exsA* mutants, *popB* mutants or wild-type PAO1 with (LT+) or without (LT-) LysoTracker staining was quantified (Figure 2). For *exsA* mutants, the mean number of bacterial-occupied LT (+) vacuoles per cell was 3.8 +/- 0.3, significantly more than the number of bacterial-occupied LT (-) vacuoles at 1.9 +/- 0.3 [p < 0.001 Welch’s corrected t-Test] (Figure 2A). The *popB* mutant was more likely to occupy LT (-) vacuoles than the *exsA* mutant with 3.6 +/- 0.7 bacteria-occupied LT (-) vacuoles per cell versus 1.9 +/- 0.3 for the *exsA* mutant [p < 0.05, Welch’s corrected t-Test] (Figure 2A). Indeed, the *popB* mutant was located exclusively in LT (-) vacuoles in ~13% of all infected epithelial cells versus ~2% for the *exsA* mutant [p = 0.01, Chi-square test]. There was no significant difference in the numbers of LT (+) and LT (-) bacteria-occupied vacuoles per cell for the *popB* mutant compared to wild-type PAO1 infected cells (Figure 2A). As would be expected, considering that wild-type PAO1, but not the *popB* mutant, can form and traffic to bleb-niches, there were significantly fewer bacterial-occupied vacuoles per cell for PAO1 compared to *popB* mutant-infected cells, regardless of LysoTracker-staining [LT (-) = 1.7 +/- 0.2 versus 3.6 +/- 0.7, respectively, p < 0.05: LT (+) = 1.9 +/- 0.2 versus 3.95 +/-0.3, respectively, p < 0.001 Welch’s corrected t-Test] (Figure 2A). From counts of individual GFP-expressing wild-type bacteria, it appeared that epithelial cells with bleb-niches contained significantly more intracellular bacteria [mean value of 7.1 +/- 1.3 bacteria per cell] than non-blebbing cells [2.4 +/- 0.3 bacteria per cell, p = 0.002 Welch’s corrected t-Test].

LT (+) vacuoles occupied by the *popB* and *exsA* mutants were found at a similar frequency [mean value of 3.95 +/- 0.3 per cell versus 3.8 +/- 0.3 per cell, respectively, p = 0.79, Welch’s corrected t-Test] (Figure 2A). To normalize for differences in bacterial internalization between these two mutants, the mean percentage of bacteria-occupied LT (+) vacuoles was calculated as a function of the total number of occupied vacuoles in a given cell (Figure 2B). For the *exsA* mutant most occupied vacuoles were LT (+) (73.9 +/- 2.1%), significantly more than either the *popB* mutant (52.4 +/- 5.4%, p = 0.001 Welch’s correct t-Test) or wild-type PAO1 (45.9 +/- 5.8%, p = 0.001 Welch’s corrected t-Test), which did not significantly differ from each other (p = 0.42 Welch’s corrected t-Test).

Using the same experimental conditions, *P. aeruginosa* infected epithelial cells were then examined for vacuole size and vacuole spatial localization, the latter classified as either perinuclear or otherwise (Table 2). LT (+) vacuoles occupied by the *exsA* mutant were significantly larger in diameter [1.15 +/- 0.04 µm] than the corresponding LT (-) group [0.99 +/- 0.06 µm, p < 0.05 Welch’s corrected t-Test]. The LT (+) occupied vacuoles were also more likely to be perinuclear (Table 2). PAO1 occupied LT (+) vacuoles were also significantly larger than their LT (-) counterparts, and more of those LT (+) occupied vacuoles were perinuclear, although that difference was not significant. LT (-) vacuoles occupied by the *popB* mutant were larger [1.07 +/- 0.06 µm] than those occupied by PAO1 [0.89 +/- 0.07 µm].

**Table 2:** Mean size and spatial distribution of bacteria-occupied vacuoles.

Bacterial Strain	Mean Vacuole Size (+/- SEM) µm		% Bacteria-Occupied Vacuoles which are Perinuclear [Mean (+/- SEM)]	
	Non-Acidic Vacuoles	Acidic Vacuoles	Non-Acidic Vacuoles	Acidic Vacuoles
*exsA* mutant	0.99 +/- 0.06	1.15 +/- 0.04^†^	10.9 +/- 1.5	34.3 +/- 3.7^†^
*popB* mutant	1.07 +/- 0.06^††^	1.18 +/- 0.05	12.8 +/- 2.0	24.4 +/- 5.4
PAO1 wild-type	0.89 +/- 0.07	1.10 +/- 0.05^†^	19.2 +/- 4.8	32.9 +/- 5.3

Values compared using ANOVA (p = 0.004) and pairwise using Welch’s corrected t-Test

^†^ Significant difference from non-acidic vacuoles of the same strain (p < 0.05, Welch’s corrected t-Test)

^†^^†^ Significant difference from non-acidic vacuoles of PAO1 (p < 0.05, Welch’s corrected t-Test)

Together, the data show that without the T3SS, i.e. *exsA* mutants, the majority of intracellular *P. aeruginosa* are trafficked to acidified perinuclear vacuoles within epithelial cells, while wild-type and translocon (*popB*) mutants (both able to secrete T3SS effectors including ExoS) are less likely to occupy acidified vacuoles, even though the *popB* mutant cannot translocate effectors across host membranes.

### Bafilomycin Rescues Intracellular Survival of the *exsA* Mutant

**Figure 3:**
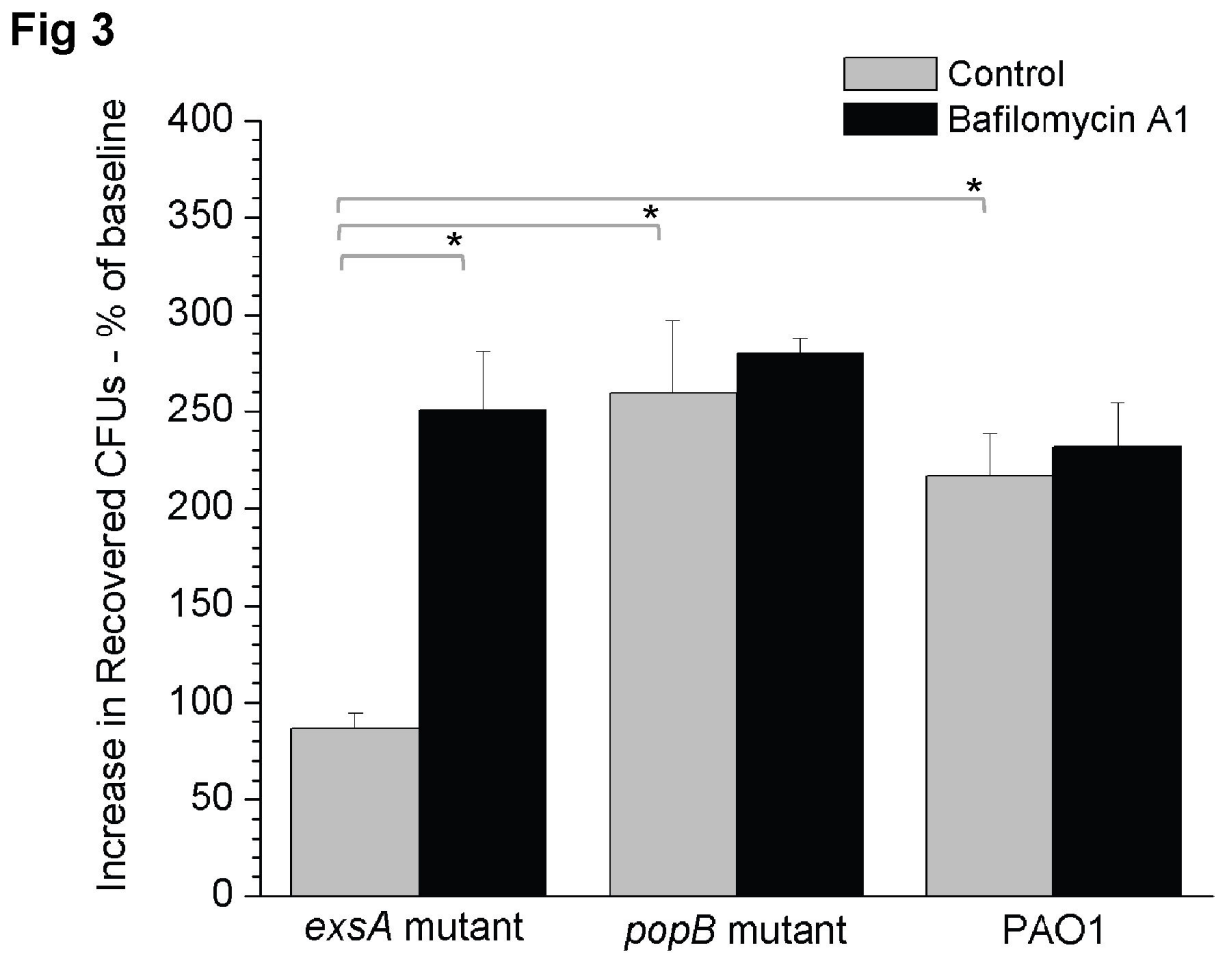
Intracellular survival and replication of *P. aeruginosa* PAO1 and its type III secretion mutants in corneal epithelial cells in the presence bafilomycin A1 (200 nM) (black boxes) versus control cells treated with vehicle only (grey boxes). Bafilomycin treatment restored intracellular survival of the *exsA* mutant to that of the *popB* mutant and wild-type PAO1. Bafilomycin A1 was added 1 h before infection and continued throughout the assay. Intracellular survival was expressed as the mean percentage increase in viable intracellular bacteria at 8 h versus 4 h post-infection (+/- SEM). A representative experiment of 3 independent experiments in shown above. ANOVA (p = 0.0002) and Welch’s corrected t-test were used for statistical analysis (* p < 0.05).

We next explored if association of the *exsA* mutant with acidified vacuoles was related to a reduced capacity to thrive intracellularly. For this purpose, intracellular survival assays were performed with and without bafilomycin A1, an inhibitor of vacuole acidification, using the *exsA* mutant. Wild-type PAO1 and the *popB* (translocon) mutant were included as controls (Figure 3). As expected, wild-type and translocon mutants replicated intracellularly, and their replication rate was unaffected by bafilomycin A1. Without bafilomycin, the *exsA* mutant was confirmed to be defective in intracellular replication [86.4 +/- 15% relative to baseline] compared to wild-type (216.8 +/- 40%) and *popB* mutant bacteria (259.6 +/- 65%, p < 0.05, Welch’s corrected t-Test). With bafilomycin A1 (200 nM) intracellular replication by the *exsA* mutant was rescued to levels similar to wild-type PAO1 (250.7 +/- 52.2%) (Figure 3). Control experiments (not shown), confirmed that 200 nM bafilomycin A1 blocked LysoTracker staining of epithelial cells and had no impact on bacterial viability. Thus, vacuolar acidification was required for cells to suppress intracellular replication by the *exsA* mutant.

### The ExoS ADP-ribosylation Domain Reduces Bacterial Occupation of Acidified Vacuoles

**Figure 4:**
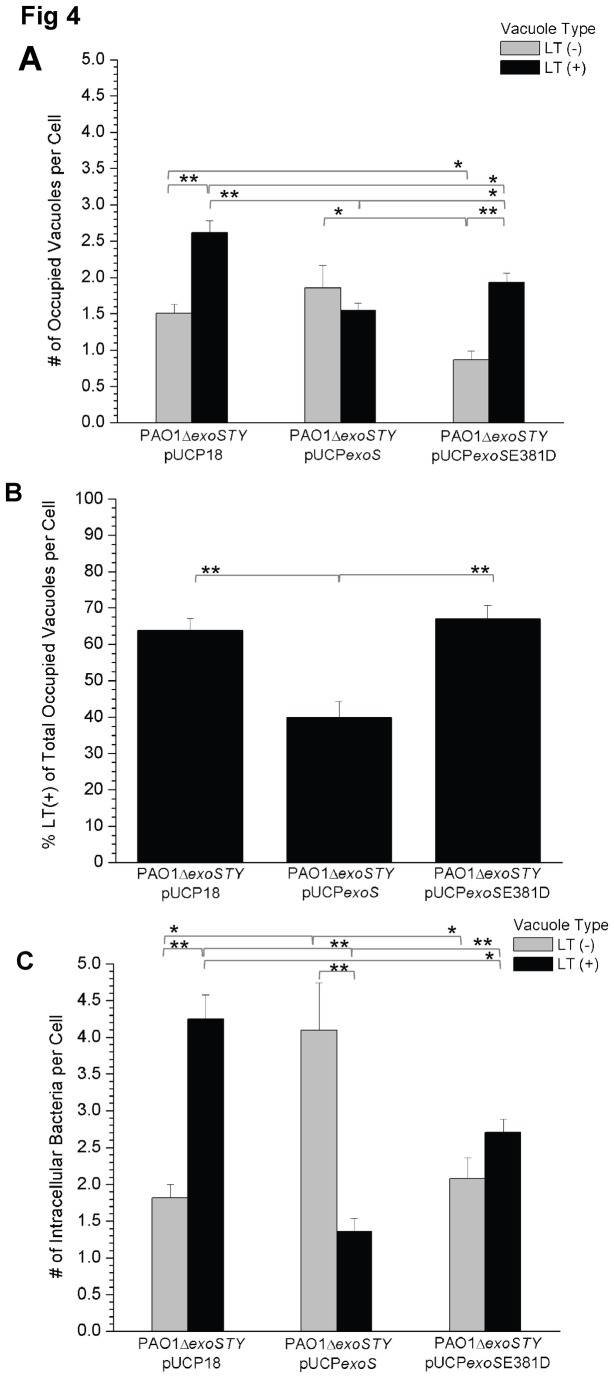
Quantification of acidified versus non-acidified vacuole occupation by a triple effector type III secretion mutant of *P. aeruginosa* complemented with either *exoS* or *exoS* without ADPr activity. (A) Confocal microscopy images were used to classify bacteria-occupied vacuoles as LysoTracker LT (+) (acidified) or LT (-) at 5 h post-infection with a triple effector mutant of *P. aeruginosa* (PAO1Δ*exoSTY*) complemented with *exoS* (pUCP*exoS*), *exoS* without ADPr activity (pUCP*exoS*E381D) or a vector control (pUCP18). Data are shown as the mean (+/- SEM) values of bacteria-occupied vacuoles per cell. Grey columns denote LT (-) vacuoles, black columns denote LT (+) vacuoles. Calculations excluded cells showing bleb-niche formation. Without ExoS ADPr activity (complementation with pUCP18 or pUCP*exoS*E381D), there were significantly more acidified bacteria-occupied vacuoles per cell (p < 0.05 Welch’s corrected t-Test). (B) The number of LT (+) bacteria-occupied vacuoles per cell was also calculated as a function of the total number of bacteria-occupied vacuoles per cell. Mean percentage (+/- SEM) is shown. Expression of ADPr active *exoS* was associated with reduced occupation of acidified vacuoles. Calculations also excluded cells showing bleb-niche formation. (C) Mean (+/- SEM) values of intracellular bacteria were determined to account for both the number of bacteria per vacuole and bacteria within blebbing cells in non-vacuolar niches. Complementation of the triple effector mutant PAO1Δ*exoSTY* with *exoS* (pUCP*exoS*) significantly reduced the number of intracellular bacteria per cell within acidified compartments. Grey columns denote LT (-) vacuoles, black columns LT (+) vacuoles. Each panel above is a representative experiment of 3 independent experiments. Significant differences were observed between groups by ANOVA (p < 0.0001). Welch’s corrected t-Test was used in pair-wise comparisons [*p < 0.05, **p < 0.001].

We previously reported that the ADPr domain of ExoS confers intracellular replication without the T3SS translocon or other known effectors [30]. Thus, we tested if this domain of ExoS impacts *P. aeruginosa* occupation of acidified vacuoles. A triple effector mutant of PAO1 (PAO1Δ*exoSTY*) complemented with *exoS* (pUCP*exoS*) was compared to the same mutant complemented with ADPr-inactive *exoS* (pUCP*exoS*E381D) and a vector control (pUCP18). The two controls occupied more LT (+) acidified vacuoles than LT (-) vacuoles (Figure 4A) [pUCP18 LT (+): 2.6 +/- 0.2 versus LT (-): 1.5 +/- 0.1, pUCP*exoS*E381D LT (+): 1.9 +/- 0.1 versus LT (-): 0.9 +/- 0.1, p < 0.001 Welch’s corrected t-Test], similar to the results for the *exsA* mutant (Figure 2A). Complementation with ADPr active ExoS reduced this bias towards bacteria-occupied LT (+) vacuoles relative to LT (-) vacuoles (Figure 4A) [LT (+): 1.5 +/- 0.1 versus LT (-): 1.6 +/- 0.2, p = 0.82 Welch’s corrected t-Test]. This was the case even after normalizing for differences in bacterial internalization, i.e. when the mean percentage of bacteria-occupied LT (+) vacuoles was calculated as a function of the total number of occupied vacuoles: PAO1Δ*exoSTY* + pUCP*exoS* (39.9 +/- 4.5%) versus PAO1Δ*exoSTY* + pUCP*exoS*E381D (67.7 +/- 3.7%) [p < 0.001 Welch’s correct t-Test], the latter was not significantly different from PAO1Δ*exoSTY* + pUCP18 (63.8 +/- 3.3%) (Figure 4B). It was noted that pUCP*exoS-*complemented bacteria partitioned exclusively to LT (-) vacuoles in 35% of the infected, non-blebbing cells versus only 10% of the cells infected with pUCP18 strain [p < 0.001 (chi-square)]. This would account for the lower overall percentage of LT (+) vacuoles per cell calculated for the *exoS*-expressing strain, despite the apparent overlap in the mean number of LT (+) versus LT (-) occupied vacuoles per cell.

To account for variation in the number of bacteria per vacuole and the ability of some bacteria to traffic to non-vacuolar compartments, the mean number of intracellular bacteria per cell was also calculated, regardless of whether bacteria occupied vacuoles or bleb-niches, and their association with LysoTracker was recorded (Figure 4C). Complementation of PAO1Δ*exoSTY* with *exoS* was associated with significantly fewer intracellular bacteria in acidified compartments [+ pUCP*exoS* LT (+): 1.4 +/- 0.2, p < 0.001 Welch’s corrected t-Test] compared to either the control plasmid [pUCP18 LT (+): 4.2 +/- 0.3] or complementation with ADPr-inactive *exoS* [+ pUCP*exoS*E381D LT (+): 2.7 +/- 0.2]. Interestingly, complementation with ADPr-inactive *exoS* also reduced bacterial occupation of LT (+) compartments compared to the control plasmid complemented mutant, but not to the reduced levels achieved by pUCP*exoS* complementation (Figure 4C). These data show that the ADPr domain of *exoS* is important in *P. aeruginosa* evasion of acidified compartments in epithelial cells after internalization.

### Transcription of *exoS* by Intracellular *P. aeruginosa* and Evasion of Acidified Vacuoles

Expression of ExoS by *P. aeruginosa* is activated in a low calcium environment or by contact with host cells [35,36]. ExoS also regulates contact-dependent T3SS expression [37]. Since our data showed that ExoS ADPr activity reduces bacterial occupation of acidified compartments, we used a transcriptional reporter to study both relative levels and spatial patterns of *exoS* expression by intracellular *P. aeruginosa*. To accomplish this, *P. aeruginosa* PAO1 was transformed with a reporter construct pJNE05 (Table 1) that expresses *gfp* under control of the *exoS* promoter [33]. They were also transformed with plasmid p67T1 (Table 1), such that they constitutively express dTomato, another fluorophore. Under non-inducing conditions, i.e. tissue culture media, nearly all of the plasmid-bearing bacteria produced detectable, but low levels of GFP [< 1000 total fluorescence intensity] (data not shown). Under T3SS-inducing conditions (i.e. low calcium media), ~ 50% of transformed PAO1 expressed GFP at levels > 1000 total fluorescence intensity. Consequently, these values were used as guidelines to classify intracellular bacteria as having a low or high *exoS* transcriptional output.

**Figure 5:**
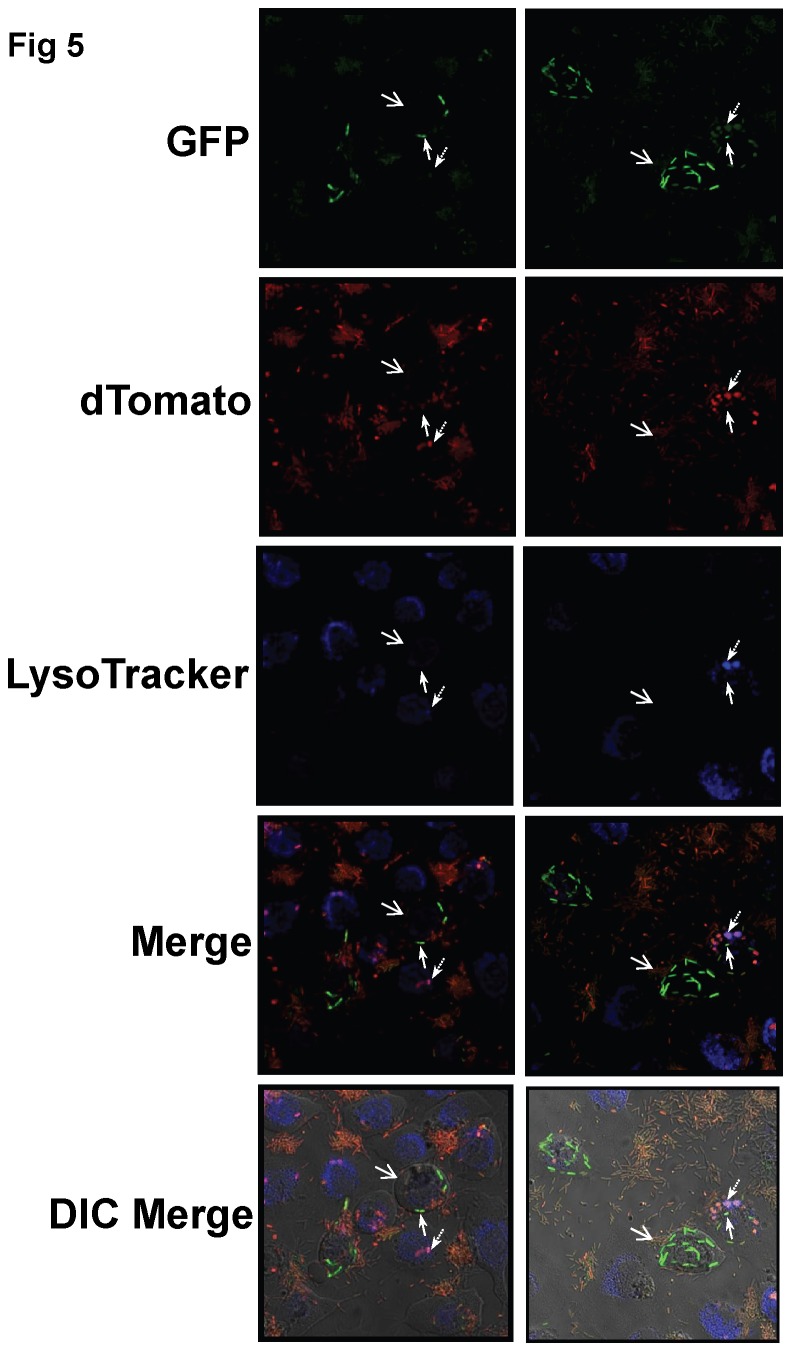
Colocalization of *P. aeruginosa* with acidified versus non-acidified vacuoles in relation to *exoS* transcriptional output. Confocal and Differential Interference Contrast (DIC) microscopy of human corneal epithelial cells at 5 h post-infection with *P. aeruginosa* PAO1 complemented with a reporter construct pJNE05 encoding the *exoS* promoter fused to *gfp* (green), and p67T1 which constitutively expresses dTomato (red). Bacteria were classified as having a high *exoS* transcriptional output using a threshold value of 1000 units of GFP fluorescent intensity (green) based on expression levels observed under T3SS-inducing conditions (see Results). Prior to imaging, epithelial cells were infused with LysoTracker DND-22 (blue). ExoS-expressing bacteria (high output, green) [solid arrows] were located primarily outside of acidified (blue) intracellular compartments, which often contained bacteria with low *exoS* output [dashed arrows]. Blebs are indicated with open arrows. Representative images are shown from two independent experiments. Magnification ~ 600 x.

**Figure 6:**
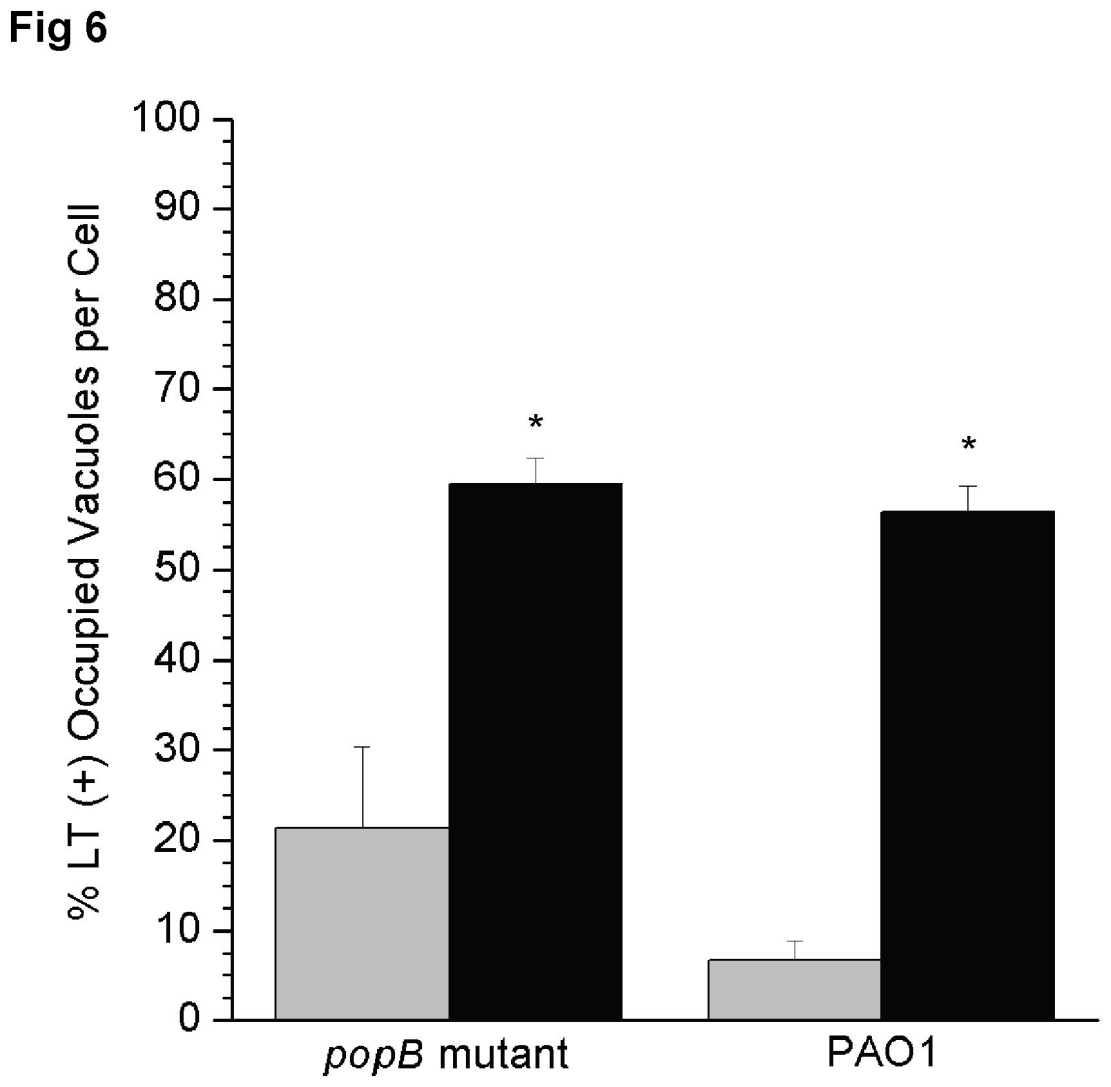
Quantification of acidified vacuole occupation by *P. aeruginosa* in relation to *exoS* transcriptional output. Data show the mean (+/- SEM) percentage of bacteria-occupied acidified (LT+) vacuoles at 5 h post-infection for *P. aeruginosa* PAO1 and a *popB* (translocon) mutant. Bacteria were transformed with an *exoS* transcriptional reporter plasmid pJNE05 (*exoS*-*gfp*) and plasmid p67T1 (*dTomato*). Infected cells were also stained with LysoTracker. Bacteria with high *exoS* expression (grey columns) were significantly less likely to occupy acidified vacuoles than those with a low *exoS* expression (black columns) (* p < 0.001, Welch’s corrected t-Test). Data is representative of 3 independent experiments.

When this transcriptional reporter strain was studied in the context of epithelial cell infection, the results show a clear distinction between intracellular bacteria with high or low *exoS* output and occupation of LT (+) vacuoles (Figure 5). High *exoS* transcriptional output coincided with low occupation of LT (+) vacuoles and *vice versa*. For example, high *exoS* transcriptional output was observed in 26 +/- 6.7% of all intracellular PAO1, very few of which occupied LT (+) vacuoles (6.6 +/- 2.2%) (Figure 6), and most were within blebbing cells. The remaining intracellular PAO1 (74 +/- 6.7% of total bacteria) displayed low level *exoS* transcription and were more likely to occupy LT (+) vacuoles (56.4 +/- 3.5%) than the high transcriptional output group (p <0.001 Welch’s corrected t-Test) (Figure 6). Similar results were obtained with the *popB* mutant transformed with the same plasmids. For example, high *exoS* transcriptional output was seen in 10.6 +/- 1.6% of all intracellular translocon mutants, which were less likely to occupy LT (+) vacuoles (21.4 +/- 9.0%) as compared to bacteria with a low *exoS* transcriptional output (59.4 +/- 3.0%, p < 0.001 Welch’s corrected t-Test) (Figure 6). Together, the data show that *exoS* expression is associated with reduced occupation of acidified vacuoles by intracellular *P. aeruginosa*.

## Discussion

The data presented in this study show that the T3SS, and specifically the ADPr activity of ExoS, redirects *P. aeruginosa* away from acidified compartments within epithelial cells that have internalized them, and that this enables intracellular replication. Thus, ExoS mutants lacking ADPr activity traffic more often to acidified compartments, where they fail to thrive.

The fact that inhibition of vacuolar acidification restored the ability of the T3SS defective *exsA* mutant to replicate intracellularly shows that the inability to thrive results from acidification of the vacuoles that they are confined within. This provides insights into the likely mechanism by which epithelial cells kill intracellular *P. aeruginosa* lacking ExoS ADPr activity; inhibiting acidification reduces the activity of acid-dependent antimicrobial factors, e.g. acid-hydrolases, within epithelial vacuoles by drug-induced elevation of vacuolar pH and/or the prevention of phagosome maturation by inhibition of lysosome fusion as shown previously for autophagosome maturation [38].

Whether ExoS inhibits vacuolar acidification directly, or by redirecting bacteria to other compartments within the cell is yet to be directly determined. Supporting the latter possibility, wild-type PAO1 traffics to membrane blebs, where they are free to replicate without suppression by intravacuolar factors. However, *popB* (translocon) mutants, which also replicate within cells in a ExoS ADPr activity-dependent fashion, do not traffic to blebs and instead replicate within vacuoles [30]. Supporting the likelihood that ExoS acts locally upon the vacuole to inhibit acidification/promote intracellular replication is our data showing that *popB* mutant infected cells, like cells infected with wild-type bacteria, harbor a larger percentage of bacterial-occupied vacuoles that are not acidified compared to cells infected with *exsA* mutants. Also supporting the probability of direct manipulation, rather than an escape mechanism, is that ExoS ADPr activity, when introduced into cells without bacteria, blocks endocytic vesicle trafficking [39].

ExoS ADPr activity acts upon multiple cellular targets [40]. For example, inhibition of endocytic vesicle trafficking and lysosomal degradation of the epidermal growth factor receptor results from ExoS ADP-ribosylation of Rab5 and Rab9 [39]. Thus, ExoS inhibition of phagosome maturation through ADP-ribosylation of Rab5, and perhaps ExoS ADPr effects on other Rab GTPases (e.g. Rab6 or Rab9) with which it is known to interact [41], could explain our results. Those effects of ExoS could help internalized bacteria remain in immature phagosomes rather than trafficking to inhibitory LAMP3+, acidified (mature) phagolysosomes. Arguing against Rabs being the critical target for this activity of ExoS ADPr activity, however, is that *popB* mutants inhibit vacuolar acidification and replicate intracellularly. These mutants remain in vacuoles and should be unable to translocate ExoS across host (vacuolar or plasma) membranes to access Rabs that are located in the cytoplasm outside of vacuoles. None of the other known targets of ExoS ADPr activity including Ras [42] and the ERM proteins (Ezrin, Radixin, Moesin) [43], however, are known to be located inside vacuoles. However, it remains possible that ExoS can escape vacuoles in a translocon-mutant background through an alternative mechanism to exert its effects on vacuole acidification.

The observation that acidified vacuoles were sometimes within membrane blebs suggests that vacuolar trafficking might also be impacted. If so, that may or may not involve ExoS. A potential mechanism could be lysosomal exocytosis, a process triggered by plasma membrane damage including that induced by a T3SS as shown for *Salmonella* spp. [44]. This results in upregulated production of vesicles/lysosomes that are then exported to the plasma membrane for repair purposes [45]. Such a mechanism could conceivably contribute to formation of membrane blebs, and might induce bacterial trafficking to them during *P. aeruginosa* infection of epithelial cells.

Some individual bacterial cells among populations of mutants lacking ExoS ADPr activity were found in LT- (non-acidified) vacuoles. While it is possible that *P. aeruginosa* uses virulence factors in addition to the T3SS to avoid trafficking to acidic compartments, it is more likely that these were bacteria in the process of being trafficked to acidic compartments and had not yet reached their destination. The data showing that bacterial-occupied LT- (non-acidified) vacuoles were located further away from the nucleus than those that were LT+ supports that there are earlier steps in trafficking, considering that mature lysosomes tend to be perinuclear. Further, the finding that LT-vacuoles containing *exsA* mutants (ExoS secretion incapable) were smaller than those containing *popB* mutants (ExoS secretion capable) suggests they represent different compartments, consistent with the fact that only the latter can replicate inside vacuoles.

Some bacteria in wild-type infected cells expressed low levels of *exoS*, as shown using an *exoS* transcriptional reporter, and as expected these tended to localize to acidic vacuoles. Low levels of ExoS expression by those bacteria might follow loss of viability because they had been trapped in acidic vacuoles. Alternatively, these could be low-level expressers among the viable population and that is why they were trafficked to acidic vacuoles. The T3SS of *P. aeruginosa* is known to be triggered by host cell contact or low calcium conditions and requires translocation of the negative regulator ExsE [36,46]. Thus, individual bacteria inside a cell could be exposed to environments with differential triggering potential. Differences in gene expression among individual bacterial cells can also occur even when the entire population is exposed to the same inducing conditions, a phenomenon referred to as bistability [47]. Bistable gene expression could provide a survival advantage considering that activities of some *P. aeruginosa* T3SS effectors (e.g. the GAP activity of ExoS and ExoT) can inhibit host cell invasion and that the desirability of being internalized by a cell can depend on the host cell type and the prevailing extracellular conditions. Whatever the case, the fact that not all bacteria within a population express the T3SS at a given time point could explain the paradox of how invasive *P. aeruginosa* invade cells when capable of expressing antiphagocytic factors, and why only some internalized bacteria escape acidic compartments.

In summary, this study shows that epithelial cells are capable of killing internalized bacteria through a process involving trafficking to acidic compartments. This includes *P. aeruginosa* when not expressing the ADPr activity of ExoS, one of its T3SS effectors. Evasion of this fate by ExoS ADPr activity correlates with suppression of acidification, a feat that it can accomplish even when bacteria are trapped inside vacuoles, and are without the translocon normally used to transport T3SS effectors across host membranes. How ExoS accomplishes this when known targets of ExoS are located within the cell cytoplasm is to be determined.
